# Association of time spent outdoors with the risk of Parkinson’s disease: a prospective cohort study of 329,359 participants

**DOI:** 10.1186/s12883-023-03499-7

**Published:** 2024-01-02

**Authors:** Ling Hu, Yisen Shi, Xinyang Zou, Zhaohui Lai, Fabin Lin, Guoen Cai, Xianghong Liu

**Affiliations:** 1 Department of Neurology, Ganzhou People’s Hospital, No.16 Meiguan Road, Zhanggong District, Ganzhou City, 341000 Jiangxi China; 2 Department of Neurology, Union Hospital, Fujian Institute of Geriatrics, Fujian Medical University, Fuzhou, 350001 China; 3 Fujian Medical University, Fuzhou, 350001 Fujian China; 4 Department of Neurosurgery, Fujian Medical University Union Hospital, Fuzhou, 350001 China

**Keywords:** Parkinson’s disease, Time spent outdoors, Age, Sex, Vitamin D

## Abstract

**Background:**

Studies on the association between time spent outdoors and the development of Parkinson’s disease (PD) are lacking, and whether this relationship differs in different subgroups (age, sex) remains unclear.

**Objective:**

We here examined the association between time spent outdoors and the incidence of PD in different seasons.

**Methods:**

This study included 329,359 participants from the UK Biobank. Data regarding hours spent outdoors during a typical day were obtained through questionnaires. Cox proportional hazard regression models were used to estimate hazard ratios (HRs) for the association between exposure to outdoors duration and PD incidence. Restricted cubic spline was used to explore the potential nonlinear relationship between time spent outdoors and PD risk. To explore the potential mechanisms of time spent outdoors effecting the risk of PD incidence, their association with serum vitamin D was further analysed separately.

**Results:**

During a median follow-up of 13.57 years, 2,238 participants developed PD. In summer, time spent outdoors > 5.0 h/day was associated with a reduced PD risk compared with ≤ 2.0 h/day (HR = 0.84, 95% CI, 0.74–0.95). In winter too, time spent outdoors > 2.0 h/day was also associated with a reduced PD risk compared with ≤ 1.0 h/day (HR = 0.85, 95% CI, 0.76–0.94). For annual average time spent outdoors, participants who went outdoors for more than 3.5 h/day had a reduced PD risk than those who went outdoors for ≤ 1.5 h/day (HR = 0.85, 95% CI, 0.75–0.96). Additionally, sex and age differences were observed in the association between time spent outdoors and the PD risk. Moreover, Time spent outdoors was observed to be positively associated with serum vitamin D levels. Compared with serum vitamin D-deficient participants, the risk of PD was reduced by 15% in the sufficient participants.

**Conclusion:**

In the total population, higher time spent outdoors was linked to a reduced PD risk. However, this association may vary among different age or sex groups.

**Supplementary Information:**

The online version contains supplementary material available at 10.1186/s12883-023-03499-7.

## Introduction

Parkinson’s disease (PD) is the second most prevalent neurodegenerative disorder, with patients developing clinical manifestations such as resting tremor, bradykinesia, rigidity, and postural gait disturbances [1]. Some studies have suggested aging, environmental factors, and genetic factors as the main risk factors for PD [2]. Pesticides, methamphetamine, and trichloroethylene exposure can increase the PD risk, whereas coffee, anti-inflammatory drugs, high uric acid levels, and physical activity have a protective effect on PD development [3]. In addition, evidence has indicated an association between serum vitamin D and risk of PD incidence and progression [4]. The major source of vitamin D for humans is outdoor sun exposure [5]; however, large longitudinal studies exploring the association between time spent outdoors and PD incidence are lacking.

Several case-control studies have provided preliminary indications that exposure to outdoors and PD are significantly correlated [6, 7]. For example, a study involving 3,819 male outdoor workers found outdoor work to be negatively correlated with PD risk [7]. However, other studies have suggested that the association between outdoor work and PD was nonsignificant [8]. Considering that the effect of outdoor exposure on PD may include the effects of multiple factors (such as the intensity of activity and work, air pollution, and the intensity or exposure time of sunlight), a simple investigation exploring the association between exposure to outdoors and PD may not provide consistent conclusions, thereby failing to offer robust guidance for PD prevention. Therefore, studies targeting specific parameters (e.g., length of time) of outdoor exposure may be required.

A review of previous studies revealed one meta-analysis reporting that exposure to sunlight for > 15 min/week is associated with a significantly lower PD risk [9]. Similarly, a case-control study that included 201 patients with newly diagnosed PD and 199 controls without neurodegenerative diseases also reported that duration of sunlight exposure is inversely associated with PD risk [10]. However, longitudinal studies investigating the relationship between the duration of exposure to outdoor and PD risk are lacking. Meanwhile, a gap in the research evidence on the association between time spent outdoors and PD incidence in individuals with different characteristics (e.g. sex, age) remains. Sunlight is one of the main sources of vitamin D, which plays an essential role in protecting nerves [11, 12]. Vitamin D deficiency is widespread in PD patients [13], and its insufficiency may be associated with an increased PD risk [14]. Meanwhile, serum 25-hydroxyvitamin D is associated with PD symptom severity [15]. Therefore, we hypothesized that serum vitamin D plays a crucial role in the association between time spent outdoors and PD incidence.

This study was conducted on the basis of the UK Biobank cohort including 329,359 participants. The study explored the relationship between time spent outdoors and PD incidence, as well as elucidated whether variations exist in the association between time spent outdoors and PD among participants of different age, sex. In addition, we evaluated the association between serum vitamin D, time spent outdoors, and PD risk.

## Methods

### Study design and participants

The UK Biobank is a population-based large-scale prospective cohort study that recruited over 500,000 individuals aged 37–73 years between 2006 and 2010. This study received ethical approval from the North West Multi-Center Research Ethics Committee, Manchester, UK (REC reference for UK Biobank: 11/NW/0382), and all participants provided written informed consent. At baseline, the demographic information, lifestyle habits, residential environment, medical health information, and biological samples of the participants were collected and evaluated. The study regularly followed up with the participants to obtain information regarding their illnesses, deaths, and specific timeframes.

**Fig. 1:**
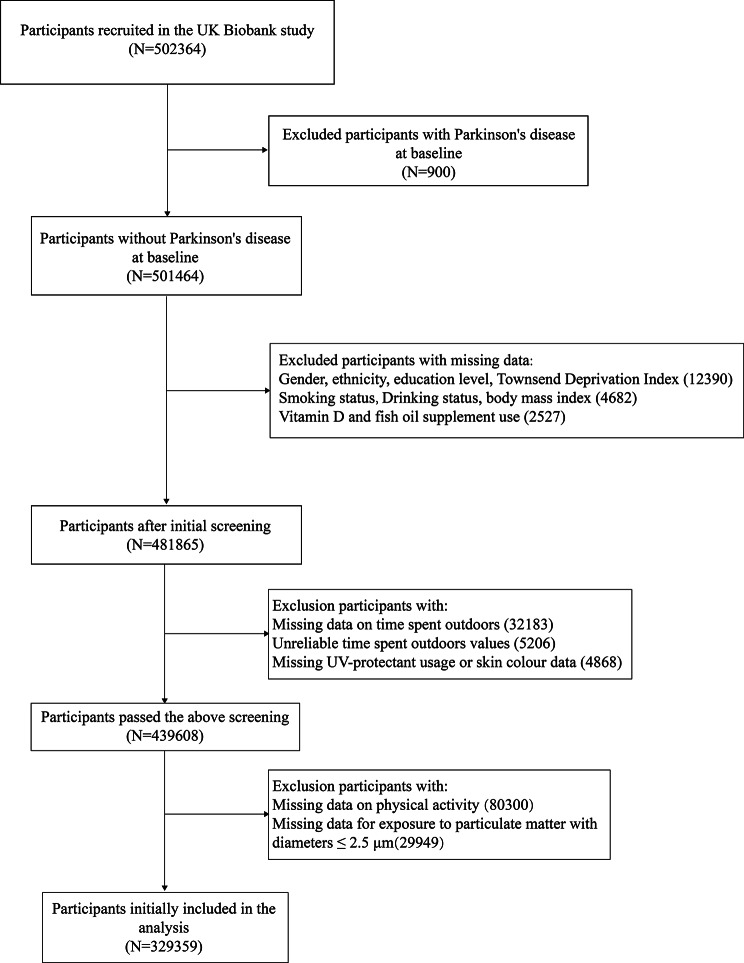
Flow chart of study design

The present study initially included 502,364 participants. From them, we excluded 900 participants with PD at baseline. Then, we excluded 19,599 participants whose data regarding sociological characteristics (gender, ethnicity, education, and Townsend deprivation index (TDI)), health-related lifestyle (smoking, drinking, vitamin D supplement use, and fish oil supplement use), or physical measurement (body mass index (BMI)) were incomplete. Next, 42,257 participants with missing data on time spent outdoors, skin colour and ultraviolet (UV) protection use, as well as those who reported extreme value of time spent outdoors, were excluded. Ultimately, 329,359 participants were included in the analysis following the exclusion of 80,300 and 29,949 participants whose data regarding physical activity status and exposure to air pollution status were missing, respectively. Based on the effective daytime hours in the UK, extremes were defined as > 16 h of time spent outdoors in summer and > 8 h of time spent outdoors in winter [16, 17]. The detailed screening process is presented in Fig. 1.

### Measurement of time spent outdoors

During the 2006–2010 baseline evaluation at the assessment center, time spent outdoors was assessed using a touchscreen questionnaire that asked “On a typical day in summer, how many hours do you spend outdoors?”; individuals could respond with a single integer number or with one of the three pre-set options: “less than 1 h/day,” “do not know,”, or “prefer not to answer.” This section was defined as “time spent outdoors in summer (fields 1050)” and “time spent outdoors in winter (fields 1060)” in the UK Biobank database. If the participant’s daily time spent outdoors varied significantly between days, an average time per day was provided. In addition, to standardize the measurement of time spent outdoors, a new variable was created: the mean of time spent outdoors in summer and winter. This variable was used to assess the average time that participants spent outdoors throughout the year.

### Measurement of outcome

The PD onset date in the UK Biobank was determined based on the algorithm recommended by the UK Biobank [18]. Disease information was obtained from the hospital admission electronic health records and death register through linkage with the Hospital Episode Statistics for England, Scottish Morbidity Records for Scotland, and Patient Episode Database for Wales. Dates and causes of death were obtained from the National Health Service (NHS) Information Centre and the NHS Scotland Central Register. The follow-up period was calculated as the time interval between the baseline assessment date and the endpoint occurrence (death, loss to follow-up, or PD diagnosis) or censoring dates (England (October 31, 2022), Scotland (August 31, 2022), and Wales (May 31, 2022)); whichever came first.

### Assessment of serum vitamin D

Serum vitamin D was measured through the chemiluminescence immunoassay (CLIA) on LIAISON XL (DiaSorin). Calibration and quality control were performed by UK Biobank. For more detailed information on serum biomarker measurement and assay performance, please refer to the UK Biobank online display (https://biobank.ndph.ox.ac.uk/ukb/ukb/docs/serum_biochemistry.pdf).

### Covariates

Referring to previous studies [16, 19], we included several covariates related to PD onset and exposure to sunlight: age, sex, education (higher and others), skin color (white and colored), use of sun/UV protectants (yes, no, no exposure to outdoor sunlight), PM_2.5_, vitamin D supplement use (yes, no), smoking status (current, previous, never), alcohol drinking status (current, never, former), TDI, BMI, hypertension (yes, no), diabetes (yes, no), and total physical activity (TPA) (< 600 metabolic equivalent task (MET)-minutes/week, ≥ 600 MET-minutes/week).

PM_2.5_ refers to particles with a diameter of ≤ 2.5 μm and PM_2.5_ levels in the air were obtained using a land use regression model for each address in 2010. Vitamin D supplement use was defined by the question: “Do you regularly take any of the following vitamin supplements?”, and if the participant answered yes to using any vitamin D supplements, then we defined the response as “yes.” Fish oil supplement use was defined by the question: “Do you regularly take any of the following? (You can select more than one answer),” and if the participant answered that he/she regularly takes fish oil supplements, then we defined the response as “yes.” The TDI is commonly used to measure the socioeconomic environment of a population and is a composite assessment index including household unemployment, housing overcrowding, lack of car ownership, and lack of home ownership. BMI was calculated by measuring the height (m) and weight (kg) of the participant at the assessment center during baseline measurements. The education level was divided into four groups: higher (college or university degree; NVQ, HND, HNC, or equivalent; other professional qualifications (e.g., nursing, teaching)), upper secondary (A levels/AS levels or equivalent), lower secondary (O levels/GCSEs or equivalent and CSEs or equivalent), and other. High blood pressure at baseline was identified on the basis of self-report, diagnosis by a doctor, and treatment with high blood pressure medication. Diabetes at baseline was identified on the basis of self-report, diagnosis by a doctor, and treatment with antidiabetic medication. TPA was measured using the total metabolic equivalent task minutes per week for all activities, including walking, moderate, and vigorous activity.

### Statistical analyses

Descriptive statistics were expressed as the mean [standard deviation (SD)] for continuous variables and as the number of participants (percentage) for categorical variables. To compare baseline characteristics between the PD-onset and non-onset groups, ANOVA was used to assess continuous variables, and chi-square tests were conducted to assess categorical variables.

We categorized the participants into four groups based on the quartiles of time spent outdoors duration and used the group less than the first quartile (25th) as a reference. Then, Cox proportional hazard regression models were used to measure the hazard ratios (HRs) and 95% confidence intervals (CIs) for the effect of duration of time spent outdoors on the PD risk in all participants. In this analysis, three models were constructed. Model 1 was adjusted for age and sex; Model 2 was further adjusted for education, use of sun/UV protectants, and PM_2.5_, in addition to the variables adjusted in Model 1; Model 3 was further adjusted for vitamin D supplement use, fish oil supplement use, smoking status, drinking status, TDI, BMI, hypertension, diabetes, TPA, and skin color, in addition to the variables adjusted in Model 2. The association between time spent outdoors and the PD risk was visualized by applying a restricted cubic spline (RCS) with 5 nodes (5th, 27.5th, 50th, 72.5th, and 95th). The duration of time spent outdoors was further incorporated as a continuous variable into the Cox proportional hazards regression models for analysis. For objects observed to have potential nonlinear correlations in the RCS, we will use the “segmented” R package to identify inflection points and perform segmented Cox proportional hazards regressions. Subgroup analyses of participants with different ages (≥ 60 years versus < 60 years), sexes were performed, and interactions were tested by adding a product term of the subgroup variable with time spent outdoors to the model. Using the likelihood ratio test, we tested an interaction by comparing models with and without the product term, and an interaction was considered to exist when *P* < 0.05. Furthermore, when an interaction was observed, we used the RCS to visualize the association between time spent outdoors and the PD risk in the different subgroups. Finally, we explored the relationship between time spent outdoors and serum vitamin D levels, and between serum vitamin D levels and the PD risk.

To further confirm our findings, sensitivity analyses were conducted to test the robustness of our results. First, we repeated the analyses after including participants with missing data on PM_2.5_ levels and physical activity. Second, we reanalyzed the association between time spent outdoors and PD risk among participants in the white ethnic group. Third, we included participants who reported extremes of time spent outdoors and reanalyzed the association between time spent outdoors and PD risk. Finally, we excluded participants who experienced PD events during the initial 2 years of follow-up, so as to avoid potential reverse causality. The proportional risk assumptions of the Cox model were tested using the Schoenfeld residual method, and the results obtained were determined to be satisfactory.

Statistical analyses were conducted using R 4.2.1 (R Foundation, Vienna, Austria). Two-sided *P* < 0.05 was considered statistically significant.

## Results

### Participant characteristics

In this study, 329,359 participants were included in the analysis, with a median follow-up period of 13.57 years. During follow-up, 2,238 participants developed PD. Table 1 summarizes the baseline characteristics of the participants according to whether they progressed to PD. Participants who developed PD had certain similar characteristics, including older age, lower education, higher obesity rates, reduced use of UV protectants, and with a history of diabetes and hypertension (*P* < 0.001).

**Table 1:** Baseline characteristics of all participants by occurrence of the Parkinson’s disease status

Variables	Overall	No incident Parkinson’s disease	Incident Parkinson’s disease	*P* value
Number of participants	329,359	327,121	2238	
Age at baseline, year, mean (SD)	56.23 (8.11)	56.19 (8.10)	62.84 (5.27)	< 0.001
**Sex, N (%)**				< 0.001
Female	172,543 (52.4)	171,762 (52.5)	781 (34.9)	
Male	156,816 (47.6)	155,359 (47.5)	1457 (65.1)	
**Education, N (%)**				< 0.001
Higher	210,856 (64.0)	209,542 (64.1)	1314 (58.7)	
Upper secondary	19,293 (5.9)	19,168 (5.9)	125 (5.6)	
Lower secondary	55,490 (16.8)	55,147 (16.9)	343 (15.3)	
Other	43,720 (13.3)	43,264 (13.2)	456 (20.4)	
**Smoking status, N (%)**				< 0.001
Current	31,960 (9.7)	31,829 (9.7)	131 (5.9)	
Previous	116,266 (35.3)	115,298 (35.2)	968 (43.3)	
Never	181,133 (55.0)	179,994 (55.0)	1139 (50.9)	
**Drinking status, N (%)**				< 0.001
Current	306,676 (93.1)	304,654 (93.1)	2022 (90.3)	
Previous	10,765 (3.3)	10,647 (3.3)	118 (5.3)	
Never	11,918 (3.6)	11,820 (3.6)	98 (4.4)	
**Skin color, N (%)**				< 0.001
White	248,726 (75.5)	246,940 (75.5)	1786 (79.8)	
Colored	80,633 (24.5)	80,181 (24.5)	452 (20.2)	
**Use of sun/UV protection, N (%)**				< 0.001
Yes	296,796 (90.1)	294,838 (90.1)	1958 (87.5)	
No	30,991 (9.4)	30,722 (9.4)	269 (12.0)	
Do not go out in sunshine	1572 (0.5)	1561 (0.5)	11 (0.5)	
**Vitamin D supplement, N (%)**				0.502
Yes	13,354 (4.1)	13,270 (4.1)	84 (3.8)	
No	316,005 (95.9)	313,851 (95.9)	2154 (96.2)	
**Use of fish oil supplements, N (%)**				< 0.001
Yes	104,200 (31.6)	103,399 (31.6)	801 (35.8)	
No	225,159 (68.4)	223,722 (68.4)	1437 (64.2)	
**Total physical activity, N (%)**				0.393
≥ 600 MET-min/week	269,756 (81.9)	267,939 (81.9)	1817 (81.2)	
< 600 MET-min/week	59,603 (18.1)	59,182 (18.1)	421 (18.8)	
**Prevalent diabetes, N (%)**				< 0.001
Yes	16,080 (4.9)	15,859 (4.8)	221 (9.9)	
No	313,279 (95.1)	311,262 (95.2)	2017 (90.1)	
**Prevalent hypertension, N (%)**				< 0.001
Yes	90,221 (27.4)	89,299 (27.3)	922 (41.2)	
No	239,138 (72.6)	237,822 (72.7)	1316 (58.8)	
**Body mass index, N (%)**				< 0.001
Healthy Weight	110,875 (33.7)	110,251 (33.7)	624 (27.9)	
Obese	75,388 (22.9)	74,843 (22.9)	545 (24.4)	
Overweight	141,454 (42.9)	140,390 (42.9)	1064 (47.5)	
Underweight	1642 (0.5)	1637 (0.5)	5 (0.2)	
Townsend deprivation index, mean (SD)	-1.51 (2.94)	-1.51 (2.95)	-1.59 (2.88)	0.178
PM_2.5_, ug/m3, mean (SD)	9.96 (1.05)	9.96 (1.05)	9.91 (1.03)	0.018
**Time spent in outdoors (average), N (%)**				< 0.001
≤ 1.5 h/day	104,973 (31.9)	104,407 (31.9)	566 (25.3)	
1.6–2.5 h/day	87,036 (26.4)	86,446 (26.4)	590 (26.4)	
2.6–3.5 h/day	61,187 (18.6)	60,736 (18.6)	451 (20.2)	
> 3.5 h/day	76,163 (23.1)	75,532 (23.1)	631 (28.2)	
**Time spent in outdoors (summer), N (%)**				< 0.001
≤ 2.0 h/day	120,904 (36.7)	120,224 (36.8)	680 (30.4)	
2.1-3.0 h/day	57,508 (17.5)	57,134 (17.5)	374 (16.7)	
3.1-5.0 h/day	89,290 (27.1)	88,616 (27.1)	674 (30.1)	
> 5.0 h/day	61,657 (18.7)	61,147 (18.7)	510 (22.8)	
**Time spent in outdoors (winter), N (%)**				< 0.001
≤ 1.0 h/day	177,496 (53.9)	176,424 (53.9)	1072 (47.9)	
1.1-2.0 h/day	80,757 (24.5)	80,160 (24.5)	597 (26.7)	
> 2.0 h/day	71,106 (21.6)	70,537 (21.6)	569 (25.4)	
**Serum 25(OH)D level, N (%)**				0.464
Deficient (< 25 nmol/L)	34,871 (10.6)	34,655 (10.6)	216 (9.7)	
Insufficient (25–50 nmol/L)	122,878 (37.3)	122,049 (37.3)	829 (37.0)	
Sufficient (> 50 nmol/L)	139,927 (42.5)	138,952 (42.5)	975 (43.6)	
Missing	31,683 (9.6)	31,465 (9.6)	218 (9.7)	
**Season of diagnosis of Parkinson’s disease, N (%)**				
Spring			534 (23.9)	
Summer			588 (26.3)	
Autumn			604 (27.0)	
Winter			512 (22.9)	

Data are n (%) and mean (SD). *P* values are derived using either variance or Chi-square test

### Association of time spent outdoors with PD

**Fig. 2:**
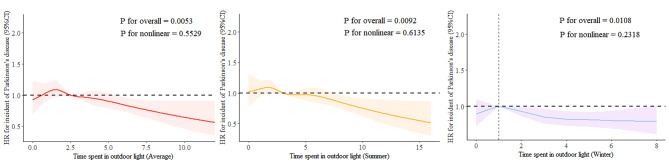
Restricted cubic spline for testing the correlation between time spent outdoors and Parkinson’s disease. Models adjusted for age, sex, education, use of sun/UV protection, skin color without tanning, PM_2.5_, vitamin D supplements use, fish oil supplements use, smoking status, drinking status, diabetes, hypertension, TDI, total physical activity, and BMI.

Table 2 presents the association between time spent outdoors and PD risk. For summer, when ≤ 2.0 h/day was used as a reference, time spent outdoors > 5.0 h/day was associated with a reduced PD risk after a range of factors were adjusted for (HR = 0.84, 95% CI, 0.74–0.95). For winter, compared with the participants who spent no more than 1 h/day outdoors, those who spent > 2 h/day outdoors exhibited a significantly reduced PD risk (HR = 0.85, 95% CI, 0.76–0.94). For the average time spent outdoors over the year, when ≤ 1.5 h/day was used as a reference, exposure times of > 3.5 h/day (HR = 0.85, 95% CI, 0.75–0.96) were associated with a reduced PD risk. Furthermore, RCS was used to illustrate the relationship between the amount of time spent outdoors and the PD risk (Fig. 2). Consistent with the findings of the Cox proportional hazards model, the RCS analyses also demonstrated that the hours spent outdoors during summer (*P* for overall = 0.0092), winter (*P* for overall = 0.0108), and on average annually (*P* for overall = 0.0053) were associated with the PD risk. When time spent outdoors was used as a continuous variable (Table 3), a 4% reduction was noted in the PD risk for every 1 h/day increase in the average annual duration (HR = 0.96, 95% CI, 0.93–0.98). In summer, a 1-h increase per day led to a 3% reduction in the PD risk (HR = 0.97, 95% CI, 0.95–0.99), while in winter, when the duration exceeded 1 h/day, each additional hour per day led to a 5% reduction in the PD risk (HR = 0.95, 95% CI, 0.92–0.98).

**Table 2:** Association between time spent outdoors and Parkinson’s disease incidence (n = 329,359)

Time spent in outdoor light	Number of incident PD/Number of participants	Model 1		Model 2		Model 3	
		HR (95%CI)	*P* Value	HR (95%CI)	*P* Value	HR (95%CI)	*P* Value
**Average**							
≤ 1.5 h/day	566/104,973	Ref		Ref		Ref	
1.6–2.5 h/day	590/87,036	0.97(0.86,1.09)	0.604	0.97(0.86,1.09)	0.601	1.00(0.89,1.12)	0.927
2.6–3.5 h/day	451/61,187	0.86(0.76,0.97)	**0.017**	0.86(0.76,0.97)	**0.016**	0.89(0.78,1.01)	0.073
> 3.5 h/day	631/76,163	0.82(0.73,0.92)	**< 0.001**	0.81(0.72,0.92)	**< 0.001**	0.85(0.75,0.96)	**0.010**
**Summer**							
≤ 2.0 h/day	680/120,904	Ref		Ref		Ref	
2.1-3.0 h/day	374/57,508	0.92(0.81,1.04)	0.191	0.92(0.81,1.04)	0.194	0.94(0.83,1.07)	0.378
3.1-5.0 h/day	674/89,290	0.87(0.78,0.97)	**0.013**	0.87(0.78,0.97)	**0.013**	0.90(0.81,1.01)	0.067
> 5.0 h/day	510/61,657	0.80(0.71,0.90)	**< 0.001**	0.80(0.71,0.90)	**< 0.001**	0.84(0.74,0.95)	**0.004**
**Winter**							
≤ 1.0 h/day	1072/177,496	Ref		Ref		Ref	
1.1-2.0 h/day	597/80,757	0.91(0.82,1.01)	0.069	0.91(0.82,1.01)	0.066	0.93(0.84,1.03)	0.165
> 2.0 h/day	569/71,106	0.83(0.75,0.92)	**< 0.001**	0.83(0.74,0.92)	**< 0.001**	0.85(0.76,0.94)	**0.002**

Model 1 adjusted for age and sex;

Model 2 further adjusted education, use of sun/UV protection, and PM_2.5_ in addition to the variables adjusted in Model 1;

Model 3 further adjusted vitamin D supplements, fish oil supplements, smoking, drinking, TDI, BMI, hypertension, diabetes, total physical activity, and skin color in addition to the variables adjusted in Model 2

Bold indicates statistical significance (*P* < 0.05)

Abbreviation: UV, Ultraviolet radiation; TDI, Townsend Deprivation Index; BMI, Body mass index

**Table 3:** Association between time spent outdoors and the risk of Parkinson’s disease (continuous variable)

Time spent in outdoor light	HR (95%CI)	*P* value
**Average**		
Increase per 1 h	0.96(0.93,0.98)	**< 0.001**
**Summer**		
Increase per 1 h	0.97(0.95,0.99)	**< 0.001**
**Winter**		
below 1 h (per 1 h increase)	1.07(0.86,1.33)	0.531
above 1 h (per 1 h increase)	0.95(0.92,0.98)	**0.003**

Model adjusted for age, sex, education, use of sun/UV protectants, PM_2.5_, vitamin D supplement use, fish oil supplement use, smoking, drinking, TDI, BMI, hypertension, diabetes, TPA, and skin color

Bold indicates statistical significance (*P* < 0.05)

Abbreviation: UV, Ultraviolet radiation; TDI, Townsend deprivation index; BMI, Body mass index; TPA, Total physical activity

### Association between time spent outdoors and PD, as stratified by sex, and age

When assessing the intricate impact of time spent outdoors on the occurrence of PD in different sex groups (Supplementary Fig. 1), a statistically significant interaction was noted between time spent outdoors in summer and sex (*P*_interaction_ = 0.0234). Based on the associations visualized using RCS (Supplementary Figs. 2 and 3), in the female population, an initial decrease was noted, followed by an increase in the PD risk with increase in summer outdoor hours (*P*_overall_ = 0.0425, *P*_nonlinear_ = 0.1128). In the male population, the PD risk exhibited a smooth and then consistent decrease with an increase in hours (*P*_overall_ = 0.0204, *P*_nonlinear_ = 0.2181). Similar significant interactions were also observed between time spent outdoors in winter and sex (*P*_interaction_ = 0.0448). Combining the results of the Cox proportional hazards model and RCS, the PD risk in the female population increased with an increase in the time spent outdoors during winter when it was < 1 h/day. However, the PD risk decreased with increasing duration when the time spent outdoors was between 1 and 3 h/day, and then exhibited an increasing trend again (*P*_overall_ = 0.0322, *P*_nonlinear_ = 0.0710). In the male population, a decreasing trend in the PD risk was noted with an increase in winter outdoor duration, although this trend was not statistically significant (*P*_overall_ = 0.1494, *P*_nonlinear_ = 0.7961). For average annual outdoor hours, the interaction with sex exhibited no significance (*P*_interaction_ = 0.2789).

The association between time spent outdoors (categorical variable) and the PD risk in participants of different age groups (≥ 60 or < 60 years) is presented in Supplementary Fig. 4. The interaction between age and average time spent outdoors annually exhibited statistical significance (*P*_interaction_ = 0.0358), while that between age and time spent outdoors in summer (*P*_interaction_ = 0.1103) and winter (*P*_interaction_ = 0.0508) exhibited no statistical significance. In younger age groups, whether in summer, winter or on an annual average, the PD risk exhibited an initial increase, followed by a decrease with an increase in outdoor hours. However, in the older age groups, a consistent trend toward reduced PD risk was noted as the number of outdoor hours increased; this was observed in summer, winter, and on the annual average. The same trend was also observed in the results presented by RCS (Supplementary Figs. 5 and 6).

### Association between serum vitamin D levels and time spent outdoors and PD risk

We observed a significant positive correlation between time outdoor hours and serum vitamin D levels (Supplementary Table 1). For the average duration of time spent outdoors, serum vitamin D levels increased by 6.04 nmol/L in participants who reported the highest duration compared with those who reported the lowest duration (β = 6.04, 95% CI, 5.83–6.24). Regarding the association of serum vitamin D levels and the PD risk (Supplementary Table 2), after a range of factors were adjusted for, a 15% reduction in the PD risk was noted in the sufficient population compared with the deficient population (HR = 0.85, 95% CI, 0.73–0.99).

### Sensitivity analysis

After we included the participants whose PM_2.5_ or physical activity status data were missing, results similar to those of previous analyses were noted (Supplementary Table 3). All participants who reported the highest number of outdoor hours exhibited a significant reduction in the PD risk compared with those who reported the lowest number of outdoor hours. This observation was consistent across hours spent outdoors during summer (HR = 0.81, 95% CI, 0.73–0.90), winter (HR = 0.83, 95% CI, 0.76–0.91), and on average annually (HR = 0.81, 95% CI, 0.73–0.90). In the analyses conducted within the white population, the same association trend was observed (Supplementary Table 4). When including people who reported extreme time spent outdoors, similar associations were observed (Supplementary Table 5). Furthermore, we obtained nearly identical results to the previous analyses when participants who had developed PD in the 2 years leading up to the follow-up were excluded (Supplementary Table 6).

## Discussion

This study focused on the association between the duration of outdoor exposure and the PD risk, while also exploring the variations in this association across different age, sex. In the total population, hours spent outdoors were negatively associated with the PD risk in summer or on average annually. For winter, a statistically significant negative trend was observed when time spent outdoors was > 1 h/day.

Time spent outdoors is associated with a range of factors, such as length of exposure to natural light (associated with circadian rhythm), levels of exposure to UV (associated with Vitamin D), and levels of exposure to fresh air. In the analysis of total population comprising 329,359 participants, negative associations were observed between the time spent outdoors and the PD risk in both the summer and on average annually. This direction of association was consistent with that noted in a meta-analysis of 3 case-control studies (295 patients with PD and 200 controls). This meta-analysis exhibited a 50-fold increased PD risk in the group that lacked exposure to sunlight compared with the group exposed to sunlight for ≥ 15 min/week (OR = 0.02, 95% CI, 0.00–0.10) [9]. In terms of effect size, different from the previous meta-analyses based on case-control studies, in this cohort study, we noted that the PD risk in participants with the highest average outdoor exposure duration (median: 5 h/day) was only reduced by 15% compared with that in those with the lowest duration (median: 1.25 h/day). Furthermore, a meta-analysis of 5,690 patients with PD and 21,251 matched controls revealed that people who work outdoors have a lower risk of developing PD [20], which further supports our findings. Moreover, UV exposure levels have been reported to be associated with PD risk. In a French population, reasonable UV radiation B (UV-B) exposure in a younger age group was associated with a lower incidence of PD [21]. This also supported our findings.

Exposure to UV-B from sunlight causes the skin to produce D3 from 7-dehydrocholesterol, which is among the primary ways through which humans obtain vitamin D [5, 22, 23]. In another study, simulated sunlight exposure significantly increased vitamin D levels in people with vitamin D deficiency [24]. Serum vitamin D levels in our study also increased with longer hours spent outdoors. Moreover, because the UK Department of Health and Social Security has consistently rejected recommendations to add vitamin D to foods such as milk, bread, and orange juice [16], sunlight was considered to be the most important resource of vitamin D in the UK population. Many studies have explored the association between vitamin D levels and PD. A study based on the results of Mini-Finland Health Survey reported that people with the highest quartile of serum vitamin D levels had a 67% reduced risk of PD compared with those with the lowest quartile (RR = 0.33, 95% CI, 0.14–0.80) [25]. Our analysis of serum vitamin D levels and the PD risk within this study population also provided preliminary evidence of a 15% reduction in the risk among participants with sufficient serum vitamin D levels compared with those with deficient levels. Regarding PD severity, a Japanese population-based study assessed PD symptoms by using the Hoehn and Yahr (H&Y) scale and Unified Parkinson’s Disease Rating Scale (UPDRS) scores. In that study, H&Y staging tended to decrease as 25-hydroxyvitamin D [25(OH)D] levels increased, while UPDRS scores exhibited a linear decrease [26]. The UPDRS offers a comprehensive, efficient, and adaptable approach for monitoring PD-associated disabilities and impairments [27]. The H&Y staging scale can be used to roughly stage disease severity, which is classified into stages 1–5 according to the PD symptoms and severity of the patients. In this scale, the early stage of PD is referred to as H&Y 1–2, the middle stage is referred to as H&Y 3–4, and the late stage is referred to as H&Y 5 [28]. Current findings have suggested that 25(OH)D may influence PD onset and severity through mechanisms such as attenuating oxidative stress and neuroinflammation, or reducing nigrostriatal dopaminergic neuronal degeneration [4]. Accordingly, we hypothesized that the effect of time spent outdoors on PD is related to its effect on change in serum 25(OH)D levels. Additionally, increased time spent outdoors was significantly associated with fewer insomnia symptoms in a study based on the UK Biobank participants [29]. Considering one study noted that decreased sleep quality and shorter duration of sleep may serve as predictive markers of PD development [30], we hypothesized that increased time spent outdoors reduces the PD risk by improving insomnia. Moreover, increased time spent outdoors was accompanied by reduced levels of exposure to artificial light at night, a risk factor associated with diseases such as depressive disorders and breast cancer [31, 32]. Artificial light has also been found to be potentially harmful for dopaminergic neurons and may be a risk factor for PD [33]. On this basis, we hypothesized that increased time spent outdoors, which is associated with a reduced PD risk, is also associated with reduced levels of exposure to artificial light.

Among individuals aged < 60 years, RCS revealed an “inverted U-shaped” correlation between outdoor time and PD, although the correlation was not statistically significant. Regarding the anomalous trend of positive correlation between time spent outdoors and the PD risk in the first half of the curve, this may be because some of the younger age groups preferred to select the exercise type that is suitable for indoor activities and has an ameliorative effect on PD (e.g., pilates, treadmill exercise), which reduced the PD risk despite the lower amount of time spent outdoors. In a meta-analysis regarding the efficacy of different exercise types on postural instability in PD patients, pilates exhibited a favorable efficacy [34]. A series of studies also showed that treadmill exercise improves gait performance in patients with mild-to-moderate PD [35]. The results of the aforementioned studies provide preliminary support for our hypothesis. Furthermore, based on the aforementioned hypothesis, we suggested that the “inverted U-shaped” trend observed between time spent outdoors and the PD risk in winter, when analyzing the overall population, is related to a greater tendency for the participants to select indoor sports during this season.

In addition, in the female population, time spent outdoors was significantly associated with PD incidence only when the duration was within a lower range. Differences in the association between time spent outdoors and disease risk across sexes were also noted in a study examining the risk of dementia [16]. A possible explanation for this phenomenon is that clothing habits of men and women often lead to differences in the skin area being exposed to sunlight, which in turn influences the degree to which sunlight affects the body. Overexposure to sunlight can cause an increased risk of melanoma [36] and cataracts [37]. Numerous studies have demonstrated a complex interrelationship between melanoma and PD [38]. Besides, A cohort study found a significantly increased PD risk in patients with cataracts compared with the normal population [39]. Based on the aforementioned studies, we hypothesized that excessively sunlight exposure in women than in men may transform light exposure from being a preventive factor for PD to a potential facilitator of PD.

This is the first large cohort study exploring the relationship between time spent outdoors and PD risk. However, this study has some limitations. First, the UK Biobank only recorded the duration of outdoor exposure, while data regarding the intensity of light and the local temperature were unavailable. Therefore, more details need to be considered in future studies. Second, because this study was only based on the UK population, its findings cannot be generalized to all populations, given that the variation in latitudes across different regions and countries causes considerable variation in UV intensity. Third, outdoor exposure hours were self-reported by the participants, and inaccurate reporting of results may have also occurred. Fourth, given that the high proportion of UK Biobank participants were in the white and affluent groups, the generalizability of the study findings will need to be validated in future studies. Finally, the UK Biobank did not provide information on specific periods spent outdoors. Data on specific periods spent outdoors may help us to further comprehend the etiology of the disease, and so, additional studies are warranted to explore this association in the future.

## Conclusion

The present study found that increased time spent outdoors was associated with a reduced PD risk in the total population. In addition, a sex and age based difference was noted in the association between time spent outdoors and the PD risk. The present study also observed that serum vitamin D levels increased as the number of hours spent outdoors increased. Also, sufficient serum vitamin D levels were associated with a decreased risk of PD. The results of this study will contribute to the development of more comprehensive PD prevention strategies. However, further studies are still needed to validate this association and discover the underlying mechanisms in this association.

### Electronic supplementary material

Below is the link to the electronic supplementary material.

Supplementary Material 1

## Data Availability

The data used in this study are available from the UK Biobank Project, access to the data is required to go through a registration and application process. For more information, please visit: https://www.ukbiobank.ac.uk/.

## Declarations

### Statement

All methods were performed in accordance with the relevant guidelines and regulations.
