# Supplementary material for: Association of time spent outdoors with the risk of Parkinson’s disease: a prospective cohort study of 329,359 participants
Source: BMC Neurol. 2024 Jan 2;24:10. doi: 10.1186/s12883-023-03499-7 (PMC10759452; doi:10.1186/s12883-023-03499-7)
Supplement: Supplementary file 1 — Supplementary Material 1 [file 12883_2023_3499_MOESM1_ESM.docx]

**Supplementary Table 1**. Association between time spent in outdoor and serum vitamin D level (N = 297676).

| Time spent in outdoors | Multivariate model | |
| --- | --- | --- |
|  | β (95%CI) | *P* value |
| Average |  |  |
| ≤1.5 h/day | Ref |  |
| 1.6-2.5 h/day | 2.41 (2.22, 2.60) | <0.001 |
| 2.6-3.5 h/day | 4.08 (3.87, 4.30) | <0.001 |
| >3.5 h/day | 6.04 (5.83, 6.24) | <0.001 |
| Summer |  |  |
| ≤2.0 h/day | Ref |  |
| 2.1-3.0 h/day | 2.12 (2.33, 1.91) | <0.001 |
| 3.1-5.0 h/day | 3.88 (3.69, 4.06) | <0.001 |
| >5 h/day | 6.04 (5.83, 6.25) | <0.001 |
| Winter |  |  |
| ≤1.0 h/day | Ref |  |
| 1.1-2.0 h/day | 2.22 (2.04, 2.40) | <0.001 |
| >2.0 h/day | 4.12 (3.93, 4.31) | <0.001 |

Model adjusted for age, sex, education, use of sun/UV protection, vitamin D supplement, fish oil supplement, smoking, drinking, TDI, BMI, hypertension, diabetes, physical activity, PM_2.5_, and skin color.

Abbreviation: UV, Ultraviolet radiation; TDI, Townsend Deprivation Index; BMI, Body mass index.

**Supplementary Table 2**. Association between serum vitamin D level and Parkinson’s disease incidence (N = 297676).

| Serum vitamin D level | Event/total | Model 1 | | Model 2 | |
| --- | --- | --- | --- | --- | --- |
|  |  | HR (95%CI) | *P* value | HR (95%CI) | *P* value |
| Deficient (<25 nmol/L) | 216/34871 | Ref |  | Ref |  |
| Insufficient (25-50 nmol/L) | 829/122878 | 0.88(0.76,1.03) | 0.106 | 0.92(0.79,1.07) | 0.267 |
| Sufficient (>50 nmol/L) | 975/139927 | 0.79(0.68,0.91) | **0.002** | 0.85(0.73,0.99) | **0.039** |
| Continuous | 2020/297676 | 1.00(0.99,1.00) | **<0.001** | 1.00(1.00,1.00) | **0.044** |

Model 1 adjusted for age, sex

Model 2 further adjusted for education, use of sun/UV protection, vitamin D supplement, fish oil supplement, smoking, drinking, TDI, BMI, hypertension, diabetes, physical activity, PM_2.5_, and skin color.

Abbreviation: UV, Ultraviolet radiation; TDI, Townsend Deprivation Index; BMI, Body mass index.

**Supplementary Table 3**. Association between time spent in outdoor and Parkinson's disease incidence after including participants with physical activity status and PM_2.5_ exposure deficits (N = 439608)

|  |  |  |  |  |  |
| --- | --- | --- | --- | --- | --- |
| Time spent in outdoor light | Number of incident PD/Number of participants | Model 1 | | Model 2 | |
| Average |  | HR (95%CI) | *P* Value | HR (95%CI) | *P* Value |
| ≤ 1.5 h/day | 755/137196 | Ref |  | Ref |  |
| 1.6-2.5 h/day | 789/115413 | 0.96(0.86,1.06) | 0.366 | 0.97(0.88,1.07) | 0.531 |
| 2.6-3.5 h/day | 617/82292 | 0.86(0.77,0.96) | **0.005** | 0.87(0.78,0.97) | **0.013** |
| >3.5 h/day | 859/104707 | 0.80(0.73,0.89) | **<0.001** | 0.81(0.73,0.90) | **<0.001** |
| Summer |  |  |  |  |  |
| ≤ 2.0 h/day | 906/157991 | Ref |  | Ref |  |
| 2.1-3.0 h/day | 512/75937 | 0.93(0.84,1.04) | 0.210 | 0.95(0.85,1.06) | 0.340 |
| 3.1-5.0 h/day | 908/120738 | 0.85(0.78,0.94) | **<0.001** | 0.87(0.79,0.95) | **0.003** |
| >5 h/day | 694/84942 | 0.79(0.71,0.88) | **<0.001** | 0.81(0.73,0.90) | **<0.001** |
| Winter |  |  |  |  |  |
| ≤ 1.0 h/day | 1441/235401 | Ref |  | Ref |  |
| 1.1-2.0 h/day | 792/107008 | 0.90(0.83,0.98) | **0.020** | 0.90(0.83,0.99) | **0.025** |
| >2.0 h/day | 787/97199 | 0.84(0.76,0.91) | **<0.001** | 0.83(0.76,0.91) | **<0.001** |

Model 1 adjusted for age and sex;

Model 2 further adjusted education, use of sun/UV protection, vitamin D supplement, fish oil supplement, smoking, drinking, TDI, BMI, hypertension, diabetes, and skin color in addition to the variables adjusted in Model 1.

Bold indicates statistical significance (*P* value <0.05).

Abbreviation: UV, Ultraviolet radiation; TDI, Townsend Deprivation Index; BMI, Body mass index.

**Supplementary Table 4**. Association between time spent in outdoor and Parkinson's disease incidence in white ethnic background participants (N = 314136)

|  |  |  |  |  |  |
| --- | --- | --- | --- | --- | --- |
| Time spent in outdoor light | Number of incident PD/Number of participants | Model 1 | | Model 2 | |
| Average |  | HR (95%CI) | *P* Value | HR (95%CI) | *P* Value |
| ≤ 1.5 h/day | 545/99340 | Ref |  | Ref |  |
| 1.6-2.5 h/day | 573/83422 | 0.97(0.86,1.09) | 0.557 | 0.99(0.88,1.12) | 0.888 |
| 2.6-3.5 h/day | 440/58645 | 0.86(0.75,0.97) | **0.016** | 0.89(0.78,1.01) | 0.075 |
| >3.5 h/day | 618/72729 | 0.82(0.73,0.92) | **<0.001** | 0.85(0.75,0.97) | **0.012** |
| Summer |  |  |  |  |  |
| ≤ 2.0 h/day | 651/114360 | Ref |  | Ref |  |
| 2.1-3.0 h/day | 363/54987 | 0.92(0.81,1.05) | 0.206 | 0.95(0.83,1.08) | 0.419 |
| 3.1-5.0 h/day | 661/85583 | 0.88(0.79,0.98) | **0.020** | 0.91(0.82,1.02) | 0.106 |
| >5 h/day | 501/59206 | 0.81(0.72,0.91) | **<0.001** | 0.84(0.75,0.96) | **0.007** |
| Winter |  |  |  |  |  |
| ≤ 1.0 h/day | 1047/169243 | Ref |  | Ref |  |
| 1.1-2.0 h/day | 577/77460 | 0.89(0.81,0.99) | **0.033** | 0.91(0.82,1.01) | 0.088 |
| >2.0 h/day | 552/67433 | 0.82(0.74,0.91) | **<0.001** | 0.84(0.75,0.93) | **0.001** |

Model 1 adjusted for age and sex;

Model 2 further adjusted education, use of sun/UV protection, vitamin D supplement, fish oil supplement, smoking, drinking, TDI, BMI, hypertension, diabetes, physical activity, PM_2.5_, and skin color in addition to the variables adjusted in Model 1.

Bold indicates statistical significance (*P* value <0.05).

Abbreviation: UV, Ultraviolet radiation; TDI, Townsend Deprivation Index; BMI, Body mass index.

**Supplementary Table 5**. Association between time spent in outdoor and Parkinson's disease incidence after including participants with extreme exposure (N = 332674).

|  |  |  |  |  |  |  |  |
| --- | --- | --- | --- | --- | --- | --- | --- |
| Time spent in outdoor light | Number of incident PD/Number of participants | Model 1 | | Model 2 | | Model 3 | |
| Average |  | HR (95%CI) | *P* Value | HR (95%CI) | *P* Value | HR (95%CI) | *P* Value |
| ≤1.5 h/day | 566/104973 | Ref |  | Ref |  | Ref |  |
| 1.6-2.5 h/day | 590/87036 | 0.97(0.86,1.09) | 0.609 | 0.97(0.86,1.09) | 0.598 | 0.99(0.88,1.12) | 0.916 |
| 2.6-3.5 h/day | 451/61187 | 0.86(0.76,0.97) | **0.018** | 0.86(0.75,0.97) | **0.016** | 0.89(0.78,1.01) | 0.068 |
| >3.5 h/day | 652/79478 | 0.82(0.73,0.92) | **<0.001** | 0.81(0.72,0.92) | **<0.001** | 0.85(0.75,0.96) | **0.008** |
| Summer |  |  |  |  |  |  |  |
| ≤2.0 h/day | 680/120982 | Ref |  | Ref |  | Ref |  |
| 2.1-3.0 h/day | 374/57536 | 0.92(0.81,1.04) | 0.194 | 0.92(0.81,1.04) | 0.196 | 0.94(0.83,1.07) | 0.376 |
| 3.1-5.0 h/day | 676/89376 | 0.87(0.78,0.97) | **0.015** | 0.87(0.78,0.97) | **0.014** | 0.90(0.81,1.01) | 0.071 |
| >5.0 h/day | 529/64780 | 0.81(0.72,0.90) | **<0.001** | 0.80(0.71,0.90) | **<0.001** | 0.84(0.74,0.94) | **0.004** |
| Winter |  |  |  |  |  |  |  |
| ≤1.0 h/day | 1072/177506 | Ref |  | Ref |  | Ref |  |
| 1.1-2.0 h/day | 597/80766 | 0.91(0.82,1.01) | 0.070 | 0.91(0.82,1.01) | 0.064 | 0.93(0.84,1.03) | 0.155 |
| >2.0 h/day | 590/74402 | 0.83(0.75,0.92) | **<0.001** | 0.83(0.75,0.92) | **<0.001** | 0.85(0.76,0.94) | **0.002** |

Model 1 adjusted for age and sex; Model 2 further adjusted education, use of sun/UV protection, and PM_2.5_ in addition to the variables adjusted in Model 1; Model 3 further adjusted vitamin D supplements, fish oil supplements, smoking, drinking, TDI, BMI, hypertension, diabetes, total physical activity, and skin color in addition to the variables adjusted in Model 2.

Bold indicates statistical significance (*P* < 0.05).

Abbreviation: UV, Ultraviolet radiation; TDI, Townsend Deprivation Index; BMI, Body mass index

**Supplementary Table 6**. Association between time spent in outdoor and Parkinson’s disease incidence after excluding participants who developed Parkinson's disease in previous 2 years of follow-up (N = 329316).

|  |  |  |  |
| --- | --- | --- | --- |
| Time spent in outdoor light | Number of incident PD/Number of participants | Model | |
| Average |  | HR (95%CI) | *P* Value |
| ≤ 1.5 h/day | 556/104963 | Ref |  |
| 1.6-2.5 h/day | 580/87026 | 1.00(0.89,1.12) | 0.949 |
| 2.6-3.5 h/day | 440/61176 | 0.89(0.78,1.01) | 0.063 |
| >3.5 h/day | 619/76151 | 0.85(0.75,0.96) | **0.011** |
| Summer |  |  |  |
| ≤ 2.0 h/day | 668/120892 | Ref |  |
| 2.1-3.0 h/day | 369/57503 | 0.95(0.83,1.08) | 0.422 |
| 3.1-5.0 h/day | 659/89275 | 0.90(0.80,1.01) | 0.061 |
| >5 h/day | 499/61646 | 0.84(0.74,0.94) | **0.004** |
| Winter |  |  |  |
| ≤ 1.0 h/day | 1053/177477 | Ref |  |
| 1.1-2.0 h/day | 584/80744 | 0.93(0.84,1.03) | 0.152 |
| >2.0 h/day | 558/71095 | 0.85(0.76,0.95) | **0.003** |

Model adjusted for age, sex, education, use of sun/UV protection, vitamin D supplement, fish oil supplement, smoking, drinking, TDI, BMI, hypertension, diabetes, physical activity, PM_2.5_, and skin color.

Bold indicates statistical significance (*P* value <0.05).

Abbreviation: UV, Ultraviolet radiation; TDI, Townsend Deprivation Index; BMI, Body mass index.


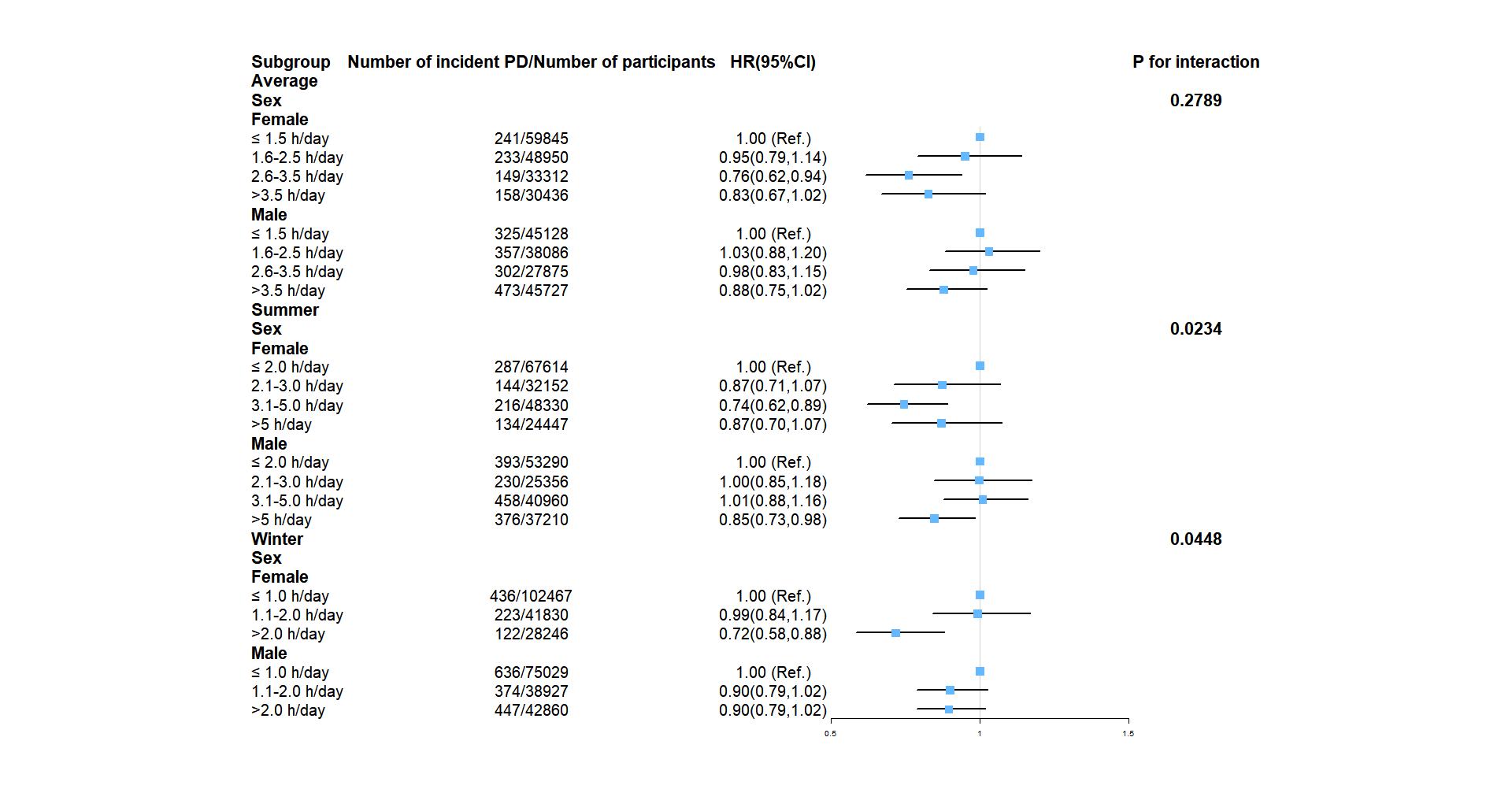


**Supplementary Fig.1.** Association between time spent outdoors and Parkinson's disease after stratification by sex. Models adjusted for age, education, use of sun/UV protection, skin colour, PM_2.5_, vitamin D supplement use, fish oil supplement use, smoking status, drinking status, diabetes, hypertension, total physical activity, TDI and BMI.


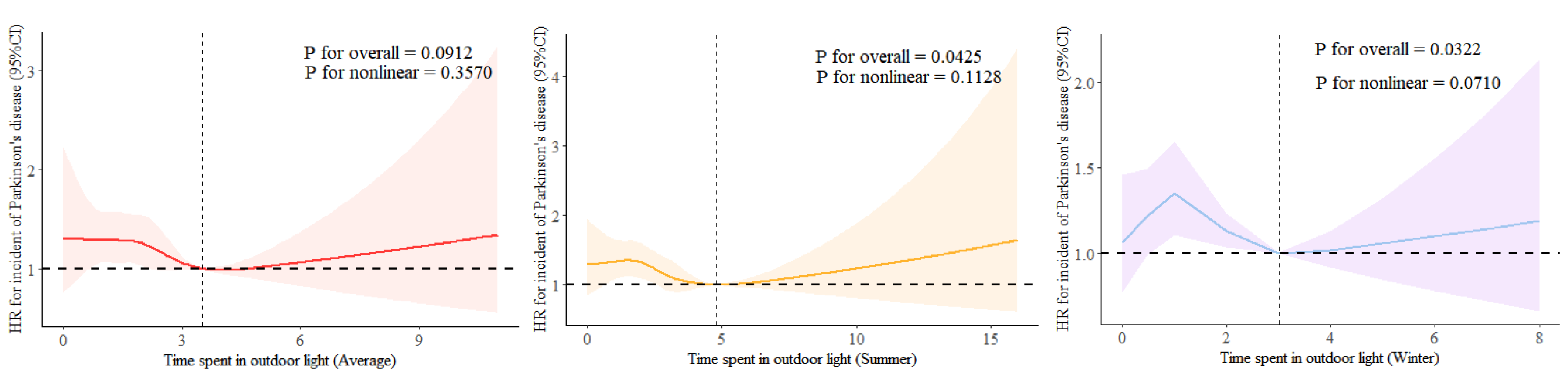


**Supplementary Fig.2.** The correlation between time spent outdoors and incident Parkinson's disease in female. Models adjusted for age, education, use of sun/UV protection, skin color, PM_2.5_, vitamin D supplements use, fish oil supplements use, smoking status, drinking status, diabetes, hypertension, TDI, BMI and total physical activity.

Abbreviation: UV, Ultraviolet radiation; TDI, Townsend Deprivation Index; BMI, Body mass index.


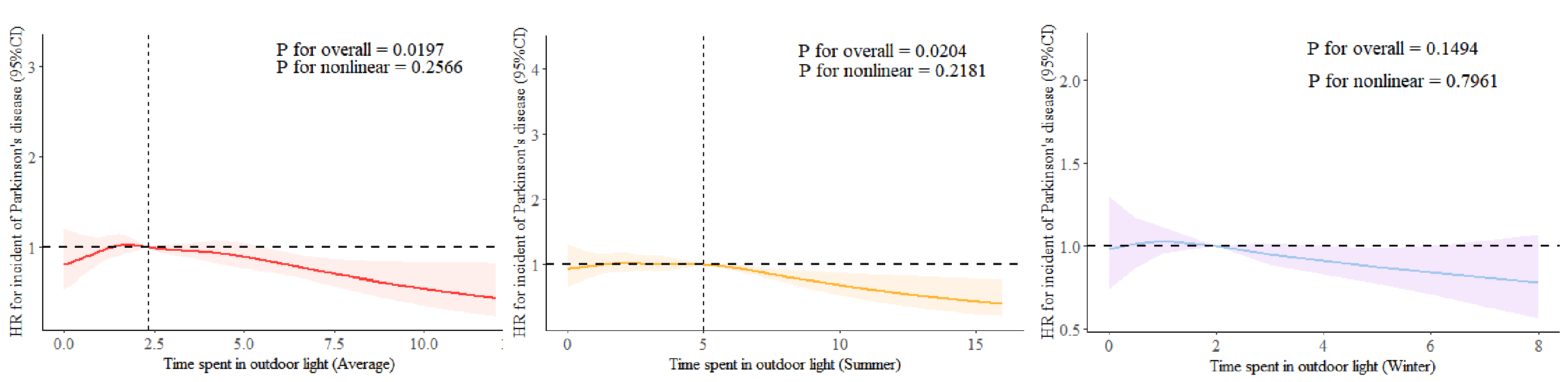


**Supplementary Fig.3.** The correlation between time spent outdoors and incident Parkinson's disease in male. Models adjusted for age, education, use of sun/UV protection, skin color, PM_2.5_, vitamin D supplements use, fish oil supplements use, smoking status, drinking status, diabetes, hypertension, TDI, BMI and total physical activity.

Abbreviation: UV, Ultraviolet radiation; TDI, Townsend Deprivation Index; BMI, Body mass index.


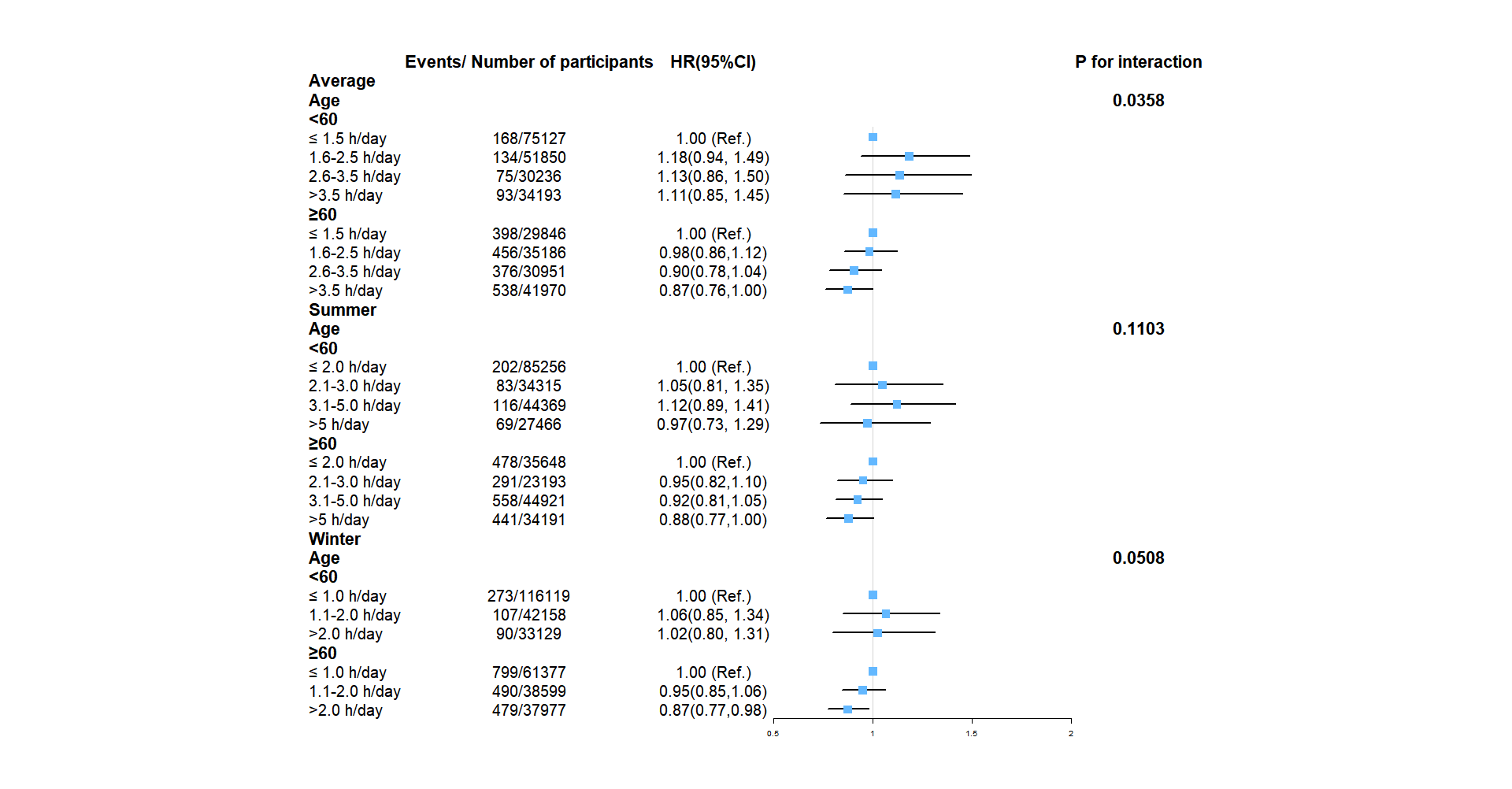


**Supplementary Fig.4.** Association between time spent outdoors and Parkinson's disease after stratification by age. Models adjusted for sex, education, use of sun/UV protection, skin colour, PM_2.5_, vitamin D supplements use, fish oil supplements use, smoking status, drinking status, diabetes, hypertension, total physical activity, TDI and BMI.


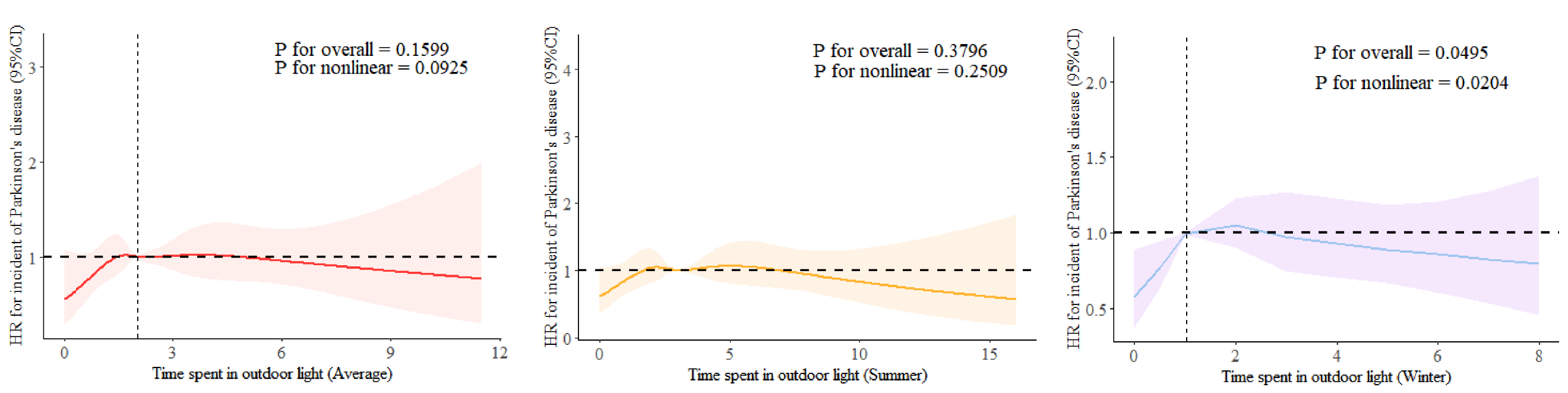


**Supplementary Fig.5.** The correlation between time spent outdoors and incident Parkinson's disease in participants with age below 60. Models adjusted for sex, education, use of sun/UV protection, skin color, PM_2.5_, vitamin D supplements use, fish oil supplements use, smoking status, drinking status, diabetes, hypertension, TDI, BMI and total physical activity.

Abbreviation: UV, Ultraviolet radiation; TDI, Townsend Deprivation Index; BMI, Body mass index.


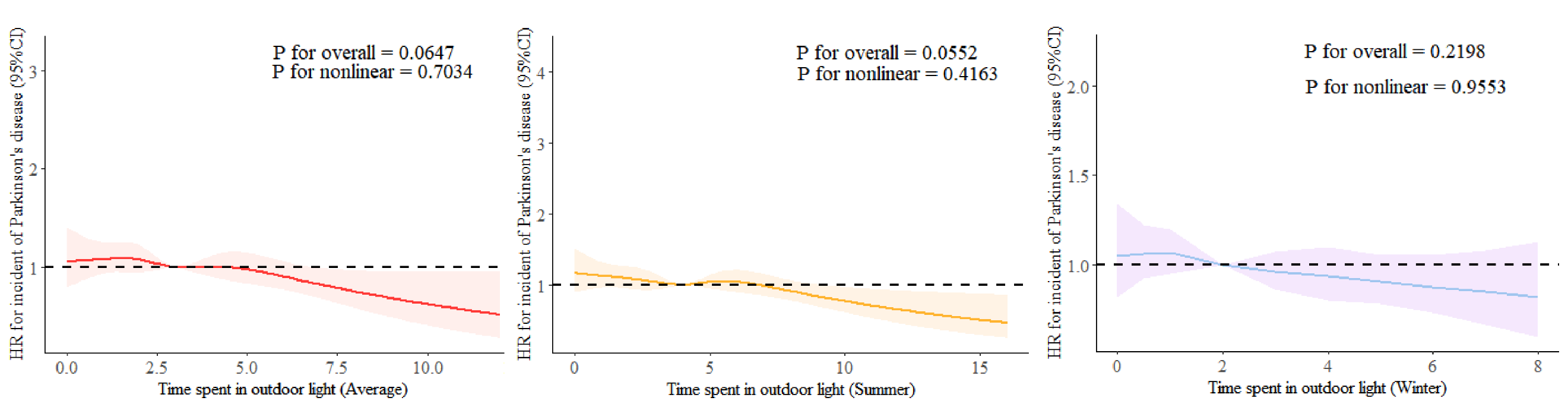


**Supplementary Fig.6.** The correlation between time spent outdoors and incident Parkinson's disease in participants with age above 60. Models adjusted for sex, education, use of sun/UV protection, skin color, PM_2.5_, vitamin D supplements use, fish oil supplements use, smoking status, drinking status, diabetes, hypertension, TDI, BMI and total physical activity.

Abbreviation: UV, Ultraviolet radiation; TDI, Townsend Deprivation Index; BMI, Body mass index.
